# Scraps

**Published:** 1889-12

**Authors:** 


### SCRAPS

PICKED UP BY THE ASSISTANT-EDITOR.

---

**The Value of Change.**—A girl out West accidentally swallowed a trade dollar a few months ago. Her life was at first despaired of; but after some six weeks' treatment and the use of a powerful specific for trade dollars, a number of red sores broke out all over her body; and when these were opened, a copper cent was found in each. At the last report eighty-four cents had been recovered in this manner, and her physician was hopeful of recovering the whole amount swallowed, perhaps with interest. —*The Medical and Surgical Reporter.*

**The Whole Truth.**—The following lines, written in the last century, are appropriate even now:

> "When quacks, as quacks may by good luck, to be sure,
> Blunder out at hap-hazard a desperate cure,
> In the prints of the day, with due pomp and parade,
> Case, patient, and doctor, are amply displayed:—
> All this is quite just—and no mortal can blame it;
> If they save a man's life, they 've a right to proclaim it:
> But there 's reason to think they might save more lives still,
> Did they publish a list of the numbers they kill!"

**Doctors' Name-Plates.**—A local practitioner, whose views are, of course, ridiculously old-fashioned, takes exception to the obtrusiveness of some of the name-plates in this neighbourhood. He says that in his earlier days they were given a modest position on the cross-portions of garden-gates or on front doors; now they are mounted high on the gates, or brought out prominently on railings. He thinks that those doctors, who are evidently so anxious to catch the eye of the passer-by, might borrow a hint from some of the shop-window arrangements that move by clock-work, put their plates still higher, and make them revolve. Fitted as a brilliant transparency, this would be particularly effective at night.

**Quelque Chose pour son Argent.**—"Il y a vraiment des clients qui sont bien exigeants," me dit mon dentiste, dont la délicatesse de sentiments ne me paraît pas valoir celle de sa main. "Je viens d'en voir un. Il en voulait pour son argent, quoi; et, à ce prix-là, il aurait voulu se faire arracher toute la mâchoire." Après avoir apaisé et calmé mon brave dentiste, je finis par apprendre que ce fameux patient qui en voulait pour son argent était un marin de passage à Paris; celui-ci se fait arracher une dent par M. R——; l'operation terminée, il demande au dentiste ce qu'il lui doit: "C'est vingt francs," répond M. R——. "Vingt francs!" s'ecrie le matelot. "Est-ce que vous vous fichez de moi? Il y a six mois, je m'en suis fait arracher une à Brest, et ça ne m'a coûté que dix sous. Pourtant le carabin de là-bas a sué pour son argent, lui; il a fait durer ça un quart d'heure et m'a trainé seize fois autour de son appartement!" Quelle poigne ils ont, les dentistes de Brest!—*L'Union medicale.*

**Chinese Doctors.**—Tcheng-Ki-Tong, a high military mandarin, has been edifying the world with some remarkable illustrations of the esteem in which native physicians are held in China. One of them having advertised that he had an infallible remedy for curvature of the spine, a hunchback applied to him, and asked if he could straighten his back. The doctor undertook to do so, and placed the unfortunate patient on his back on a flat board. He then placed a similar board on his chest and abdomen, and loaded it with heavy weights and stones. The result of this novel orthopædic surgery was, that the patient was straightened out so effectually that he died on the spot. The quack claimed his fees on the ground that he had kept his promise; the bargain was, that he should straighten his patient's back, but nothing had been said about his life! In China, it appears, the distinction between physicians and surgeons is more sharply defined than with us, and every man is expected to stick to his own branch of the profession. A rich merchant was struck by an arrow, which remained fixed in the wound. The principal surgeon of the place was sent for, and after insisting on pocketing his fee in advance cut off the projecting end of the arrow, leaving the point buried in the patient's body. On being asked to extract it, he said medical etiquette would not allow him to trepass on a brother practitioner's province; the arrow being inside the body, the case was clearly one for a physician! An old Chinaman gave the following practical advice as to how to find the most eminent doctor in a strange place: "Count the number of ghosts crouching about the doctor's doorsteps; the one most in vogue has always the largest number."—*The London Medical Recorder.*

**Doctors and Imaginative Literature.**—The contributions of medical men to the departments of imaginative work have been far from insignificant. At least four eminent members of our profession now living might be named who have found leisure, amidst absorbing occupation, so to use the pencil and brush as to gratify not only their private circles but the public, and a list of medical poets would be a long and goodly one, including such names as Akenside (the gifted singer of the pleasures of that imagination whose usefulness I am attempting to extol), Garth, Blackmore, Goldsmith, Smollett, Armstrong, Erasmus Darwin, Crabbe, Moir (better known as Delta), John Brown, whose *Rab and His Friends* is idyllic, and Oliver Wendell Holmes. Nay, even one or two of the greatest names in poetical literature might not improperly be added to such a list. Keats was apprenticed to a surgeon at Edmonton, and afterwards attended St. Thomas's Hospital. It has been argued, I am afraid not very convincingly, that Shakespeare's extensive medical knowledge proves him to have been engaged in the study of medicine during one or two of those years of his life that are unaccounted for, but it is indisputable that Dante was enrolled amongst the *medici è speziali* (leeches and druggists) of Florence, and that he attended their council meetings for several years. But it is not as producers, but as consumers of poetry and imaginative literature, that medical men derive from them their restorative influence; and as consumers they are, I feel sure, amongst the booksellers' best friends. Sydenham, when asked by Sir Richard Blackmore what course of study he would recommend for a medical student, replied, "Let him read *Don Quixote*, it is a very good book; I read it still." Conolly, the apostle of that non-restraint system to which we owe everything that is most excellent in the treatment of the insane in this country, and with which I trust professional opinion and public sentiment will permit no tampering—Conolly told me in his latter years that he took ever renewed delight in *Gulliver's Travels*. I know hard-working doctors in town and country who hold habitual converse with some of our great imaginative writers. Two of the most distinguished and busiest physicians of this day are, to my knowledge, inveterate novel readers. I have heard one of our great surgeons deliver an address betraying a deep study of the poetry of Keats; and another of our great surgeons, present at this meeting, told me recently that on his way to and from every serious operation he dips into Shelley.—SIR JAMES CRICHTON BROWNE, *in the "Address in Psychology" at the annual meeting of the British Medical Association*, 1889.

